# Functional Pathological Features and Molecular Markers in Alzheimer’s Disease

**DOI:** 10.3390/ijms27114720

**Published:** 2026-05-24

**Authors:** Mee-Na Park, Hae Won Kim, Jeong-Ho Hong, Jin Kyung Kim, Seung-Bo Lee, Hye Suk Baek, Soyoung Kwak, Ye Jin Kwon, Kibeom Park, Jieun Jeon, Na Hyeon Heo, Seong-Hun Lee, Juhyeon Cho, Shin Kim

**Affiliations:** 1 Department of Immunology, School of Medicine, Keimyung University, 1095 Dalgubeol-Daero, Dalseo-Gu, Daegu 42601, Republic of Korea; parkmn1223@gmail.com (M.-N.P.); sftwtmt@hanmail.net (H.S.B.); ourscreen3@gmail.com (K.P.); jje5726@gmail.com (J.J.); hnh9108@naver.com (N.H.H.); ss2396ss@gmail.com (S.-H.L.); wngus42930@naver.com (J.C.); 2 Department of Nuclear Medicine, Keimyung University Dongsan Hospital, 1035 Dalgubeol-Daero, Dalseo-Gu, Daegu 42601, Republic of Korea; hwkim@dsmc.or.kr (H.W.K.); ksy845@gmail.com (S.K.); kyjj0214@naver.com (Y.J.K.); 3 Department of Neurology, Keimyung University Dongsan Hospital, 1035 Dalgubeol-Daero, Dalseo-Gu, Daegu 42601, Republic of Korea; neurohong79@dsmc.or.kr; 4 Department of Microbiology, School of Medicine, Keimyung University, 1095 Dalgubeol-Daero, Dalseo-Gu, Daegu 42601, Republic of Korea; pcjlovesh6@dsmc.or.kr; 5 Department of Medical Informatics, School of Medicine, Keimyung University, 1095 Dalgubeol-Daero, Dalseo-Gu, Daegu 42601, Republic of Korea; koreateam23@kmu.ac.kr

**Keywords:** Alzheimer’s disease, neuroinflammation, neuronal cell death, synaptic dysfunction, biomarkers

## Abstract

Alzheimer’s disease (AD) is a neurodegenerative disorder defined not only by amyloid-β plaques and tau pathology but also by several interacting processes that drive disease progression. These include neuroinflammation, neuronal cell death, synaptic dysfunction, blood–brain barrier (BBB) breakdown, and myelin and axonal damage. Together, they lead to neuronal loss and cognitive decline. In this review, we present a cell-centered framework linking these processes with key molecular markers. Neuroinflammation is driven by activated microglia and astrocytes and is associated with markers such as Iba1, CD68, GFAP, and C3, along with cytokines including IL-1β and TNF-α. Neuronal cell death occurs through apoptosis, ferroptosis, pyroptosis, and necroptosis, with markers such as caspase-3, GPX4, GSDMD, and MLKL. Synaptic dysfunction is reflected by reduced synaptic proteins, including synaptophysin and PSD-95. BBB breakdown increases permeability and reduces clearance of toxic molecules. Myelin and axonal damage, associated with MBP and NfL, disrupt neural connectivity. These processes are dynamically interconnected and may contribute differently across disease stages. This integrated cell-centered and systems-level framework provides insight into AD progression while highlighting potential biomarkers and therapeutic targets for diagnosis, disease monitoring, and therapeutic intervention.

## 1. Introduction

Alzheimer’s disease (AD) is a progressive neurodegenerative disorder characterized by memory loss, cognitive decline, and behavioral changes, ultimately leading to dementia and loss of independence [1,2]. The classical neuropathological hallmarks of AD are extracellular amyloid-β (Aβ) deposits forming plaques and intracellular neurofibrillary tangles composed of hyperphosphorylated tau protein [3,4]. These lesions have traditionally been regarded as the core pathological features of the disease. However, over the past two decades, it has become increasingly evident that these features alone cannot fully account for the complexity of AD pathogenesis [5,6].

Instead, AD is increasingly viewed as a disorder involving multiple interacting functional pathological processes that converge with these primary proteinopathies to drive neurodegeneration [7,8]. Among these processes, chronic neuroinflammation has emerged as a key feature [9]. This is largely mediated by the activation of the brain’s innate immune cells, particularly microglia and astrocytes, which can adopt diverse functional states depending on the microenvironment [10,11]. Various forms of programmed cell death, including apoptosis, ferroptosis, pyroptosis, and necroptosis, have also been implicated in neuronal loss [12,13,14,15,16]. Synaptic dysfunction is another central feature, disrupting communication between neurons and correlating strongly with cognitive decline, often more closely than amyloid plaque burden [17]. In parallel, breakdown of the blood–brain barrier (BBB) compromises vascular support and permits the entry of blood-derived factors into the brain parenchyma [18,19], while degeneration of myelin and axons disrupts neural network connectivity [20,21]. These processes do not act in isolation but rather form an interconnected network that influences disease initiation, progression, and clinical presentation [8].

Understanding AD through the lens of these functional pathologies requires attention to the cellular components and their associated molecular markers. Microglial markers such as Iba1 and CD68 [22], astrocytic markers such as GFAP and C3 [10,23], and apoptotic markers such as caspase-3 activation and TUNEL staining have been widely used to assess glial activation and neuronal death [24,25]. Synaptic proteins including synaptophysin, PSD-95, and neurogranin provide insight into synaptic integrity [26,27], while BBB-associated proteins such as claudin-5, occludin, and PDGFRβ reflect vascular dysfunction [28,29]. Myelin and axonal injury can be evaluated using markers such as myelin basic protein (MBP), myelin-associated glycoprotein (MAG), and neurofilament light chain (NfL) [21,30]. These molecular signatures not only document pathological alterations but also represent potential biomarkers for diagnosis and disease monitoring, as well as targets for therapeutic intervention [31]. Functional pathology-based biomarkers therefore provide a framework for integrating multiple aspects of AD pathology [32]. Measurements of neuroinflammatory activation, neuronal injury, synaptic dysfunction, and vascular integrity offer dynamic insight into ongoing biological processes, complementing traditional Aβ and tau measures that reflect relatively static pathological deposits [24,25,33,34]. Such approaches may improve disease staging, enable prediction of clinical progression, and support the development of mechanism-based therapeutic strategies tailored to individual patients [35].

**Figure 1:**
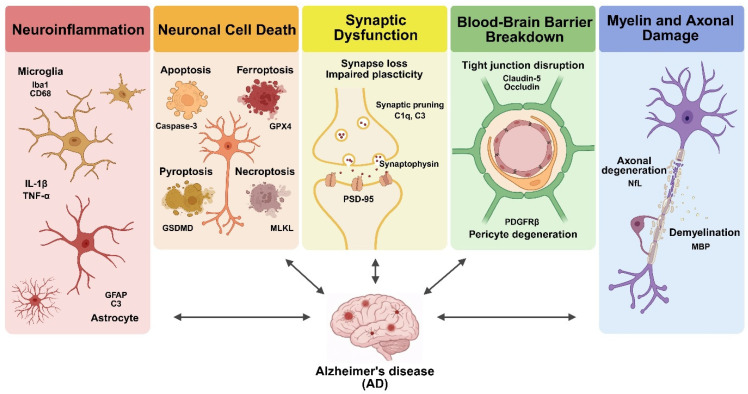
Functional pathological features and representative molecular markers in AD. Schematic overview of major functional pathological processes in AD, including neuroinflammation, neuronal cell death, synaptic dysfunction, blood–brain barrier breakdown, and myelin and axonal damage, with representative molecular markers for each process. The arrows indicate the interconnected and bidirectional relationships among these pathological processes, which collectively contribute to AD progression.

The aim of this review is to synthesize current knowledge on these functional pathological features of AD and to present them within a structured framework linking cell types, activation states, and molecular markers. We first provide an overview of key processes and representative markers, followed by focused sections on neuroinflammation, neuronal cell death, synaptic dysfunction, BBB breakdown, and myelin and axonal damage. Within each section, we discuss the relevant cell types, their functional states, and the molecular markers that characterize these states, drawing on both experimental and clinical evidence [36,37]. By integrating these perspectives, this review highlights the importance of moving beyond an amyloid- and tau-centered view of AD. A comprehensive approach incorporating biomarkers of inflammation, neurodegeneration, vascular dysfunction, and network integrity is required to capture the full complexity of the disease [38,39]. Such an approach will advance our understanding of AD pathogenesis [8] and provide a rational foundation for the development of diagnostic tools and therapeutic strategies targeting the multifactorial nature of the disorder [40]. An overview of these interconnected functional pathological processes and their representative molecular markers is summarized in Figure 1. Although previous reviews have extensively discussed individual pathological pathways and Aβ/tau pathology, the present review emphasizes a cell-centered and temporally integrated framework linking cell-type-specific pathological interactions with stage-dependent biomarker perspectives. Within this framework, vascular dysfunction, synaptic impairment, neuroinflammation, neuronal injury, and white matter degeneration are interpreted as dynamically interacting contributors to AD progression.

## 2. Functional Pathological Features and Associated Molecular Markers

### 2.1. Neuroinflammation

Neuroinflammation is a major feature of AD and is largely mediated by the activation of the brain’s innate immune cells, particularly microglia, with additional contributions from reactive astrocytes [41,42]. In AD, microglia accumulate around Aβ plaques and participate in their clearance; however, this function becomes impaired under conditions of chronic activation [43]. Sustained activation leads to the release of pro-inflammatory mediators, which are associated with neuronal injury and disease progression [44,45]. Astrocytes also undergo reactive transformation (astrogliosis), characterized by cellular hypertrophy and increased expression of intermediate filament proteins such as GFAP [46]. These glial responses are not uniform but instead represent a spectrum of activation states, reflecting the complexity of neuroinflammatory processes in AD [47,48]. Representative cell-type-specific states and molecular markers of neuroinflammation are summarized in Table 1.

#### 2.1.1. Microglial Activation and Phenotypic Diversity

In the healthy brain, microglia continuously survey the microenvironment and contribute to synaptic maintenance and debris clearance [53,54]. In AD, microglia cluster around amyloid plaques and exhibit altered morphology and function, including increased production of inflammatory mediators [55,56]. Microglial activation has historically been described using an M1/M2 polarization framework; however, this classification is now considered an oversimplification that does not capture the diversity of microglial states [43]. Instead, microglia in AD exhibit a continuum of phenotypes with overlapping functional characteristics [48]. Homeostatic microglia express markers such as P2RY12, TMEM119, Iba1, and CX3CR1 [49,57]. Upon activation, microglia can produce pro-inflammatory cytokines and reactive oxygen or nitrogen species, contributing to neuronal damage [44,49]. Elevated levels of cytokines such as interleukin-1β (IL-1β) and tumor necrosis factor-α (TNF-α) have been detected in AD brain tissue and cerebrospinal fluid (CSF), indicating an inflammatory microenvironment [50,58]. In contrast, microglia can also adopt phenotypes associated with tissue repair and debris clearance, although these functional states are not clearly separated from pro-inflammatory responses. Among the diverse activation states, disease-associated microglia (DAM) have been identified in the context of neurodegeneration. DAM are characterized by increased expression of genes involved in phagocytosis and lipid metabolism, including TREM2 and APOE, along with markers such as ITGAX, CLEC7A, and CST7 [45,48]. These cells are frequently observed in proximity to amyloid plaques and are thought to represent a response aimed at limiting plaque expansion and facilitating debris clearance [59].

Activated microglia expressing markers such as CD68 and HLA-DR are commonly found near amyloid plaques in AD brains, and their abundance has been associated with amyloid burden and disease severity [60]. In vivo imaging studies using TSPO PET further demonstrate persistent microglial activation during disease progression [61]. Aβ has also been shown to activate inflammasome pathways, including NLRP3, leading to the release of pro-inflammatory cytokines that may contribute to neuronal injury [43]. Together, these findings highlight the dynamic and context-dependent roles of microglia in AD pathology.

#### 2.1.2. Reactive Astrocytes and Phenotypic Heterogeneity

Astrocytes are key regulators of brain homeostasis and contribute to inflammatory responses in AD. Reactive astrocytes are frequently observed surrounding amyloid plaques and in regions of neuronal loss [62]. Astrogliosis is characterized by increased expression of GFAP and vimentin, along with cellular hypertrophy and, in some cases, proliferation [63]. Reactive astrocytes have been broadly classified into neurotoxic (A1-like) and neuroprotective (A2-like) states; however, this classification is increasingly recognized as an oversimplification of a heterogeneous continuum of astrocyte phenotypes [62,64]. A1-like astrocytes are typically induced by signals from activated microglia, including IL-1α, TNF-α, and complement component C1q, and are associated with the loss of normal supportive functions and the production of inflammatory mediators [10]. Markers such as GFAP, S100B, and complement component C3 have been used to identify these reactive states in AD brain tissue [51].

In contrast, astrocytes with reparative or neuroprotective features have been reported to express genes associated with synaptic support and anti-inflammatory functions, although their specific roles in AD remain less clearly defined. Recent transcriptomic studies have identified disease-associated astrocyte (DAA), which exhibit distinct gene expression profiles and increase in abundance during disease progression [52]. They co-express many “pan-reactive” genes common to reactive astrocytes (e.g., GFAP, vimentin) as well as stress and complement genes (such as Serpina3n and C3). Notably, similar DAA-like astrocytes also appear during normal aging, suggesting that age-related factors cooperate with AD pathology to drive astrocyte reactivity [52]. These astrocytes often co-express markers associated with multiple reactive states, further supporting the concept of phenotypic heterogeneity. Microglia and astrocytes interact closely and can amplify each other’s activation states. Microglia-derived cytokines promote astrocyte reactivity, while astrocyte-derived mediators can further activate microglia, creating a self-reinforcing inflammatory cycle. This bidirectional crosstalk contributes to sustained neuroinflammation and may exacerbate neuronal injury. In summary, neuroinflammation in AD is characterized by dynamic and heterogeneous activation of microglia and astrocytes. These glial responses are measurable through specific molecular markers and represent important targets for therapeutic intervention. Through interactions with synaptic dysfunction, vascular injury, and neuronal stress, sustained glial activation may contribute to the progressive amplification of AD pathology. Activated microglia and astrocytes influence neuronal and synaptic homeostasis through the release of inflammatory mediators and complement proteins, thereby linking neuroinflammation to broader neurodegenerative processes in AD [65,66,67].

### 2.2. Neuronal Cell Death

Neuronal loss is a pathological hallmark of AD and a major contributor to cognitive decline. Importantly, this neurodegeneration is not driven by a single mechanism but involves multiple forms of regulated cell death, including apoptosis, ferroptosis, pyroptosis, and necroptosis [25,68,69]. These pathways may operate in parallel and can interact through shared signaling networks, contributing to the complexity of neuronal loss and the inflammatory environment observed in AD. Major forms of regulated neuronal cell death and their representative molecular markers are summarized in Table 2.

#### 2.2.1. Apoptosis

Apoptosis is a well-characterized form of programmed cell death marked by cell shrinkage, chromatin condensation, DNA fragmentation, and membrane blebbing. It can be initiated through extrinsic signaling via death receptors or intrinsic mitochondrial stress, both of which converge on caspase activation [25,75]. In AD, evidence of apoptotic signaling has been reported in vulnerable neuronal populations. Increased expression and activation of caspases, including caspase-3, -6, and -8, have been observed in AD brains [68,70,76,77]. Cleaved caspase-3, a key executioner of apoptosis, is detected in association with neurofibrillary tangles and granulovacuolar degeneration structures rather than widespread apoptotic cell death [68]. Additional indicators of apoptosis include DNA fragmentation detected by TUNEL staining and alterations in BCL-2 family proteins, such as increased pro-apoptotic BAX and BAD and decreased anti-apoptotic BCL-2 [78]. Despite these molecular signatures, classical morphological features of apoptosis are relatively rare in AD brains, suggesting that apoptotic signaling may be incomplete or occur at a slow rate in post-mitotic neurons [79]. Experimental studies further support a role for apoptosis in AD pathology; for example, modulation of pro-apoptotic regulators such as BAD has been shown to influence neuronal survival and disease-related phenotypes in AD models [80]. Together, these findings indicate that apoptosis contributes to neuronal loss in AD, although it may operate in a modified or partial form compared with classical apoptosis observed in other cell types.

#### 2.2.2. Ferroptosis

Ferroptosis is an iron-dependent form of regulated cell death driven by lipid peroxidation. It is characterized by the accumulation of reactive oxygen species and lipid peroxides due to impaired antioxidant defenses, particularly reduced activity of glutathione peroxidase 4 (GPX4) and depletion of glutathione [81]. Morphologically, ferroptosis is associated with mitochondrial shrinkage, increased membrane density, and eventual membrane rupture, without involvement of caspases [82]. In AD, increasing evidence suggests that ferroptosis-related processes contribute to neuronal vulnerability. Postmortem and imaging studies have demonstrated iron accumulation in AD brains, particularly in the hippocampus and cortex, which correlates with markers of oxidative damage [71,83,84]. Elevated levels of lipid peroxidation products, such as 4-hydroxynonenal and malondialdehyde, have been detected in affected regions. In parallel, AD brains exhibit reduced antioxidant capacity consistent with impaired GPX4 activity, indicating a diminished ability to detoxify lipid peroxides [81]. These features—iron dyshomeostasis, lipid oxidative stress, and impaired antioxidant defenses—are consistent with ferroptosis-like mechanisms. Experimental studies further support this association. Aβ-induced toxicity has been shown to promote lipid peroxidation and iron-dependent oxidative stress in neuronal models, processes that are consistent with ferroptosis-like cell death [85]. In addition, tau pathology has been associated with oxidative stress and disruptions in iron metabolism, which may further increase neuronal susceptibility to ferroptosis [86]. Pharmacological modulation of ferroptosis pathways has shown neuroprotective effects in experimental models, including the use of lipid peroxidation inhibitors and iron chelators [72]. Moreover, early clinical observations using iron chelation strategies have suggested potential benefits in slowing cognitive decline, although further validation is required [87]. Biomarkers commonly used to assess ferroptosis in AD include lipid peroxidation products, iron accumulation, and the expression of ferroptosis-regulatory proteins such as GPX4, ferritin, transferrin receptor, and SLC7A11 [88].

#### 2.2.3. Pyroptosis

Pyroptosis is a lytic, pro-inflammatory form of programmed cell death triggered by activation of inflammasome complexes. In this pathway, inflammatory caspases, including caspase-1, cleave gasdermin family proteins such as gasdermin D (GSDMD), generating N-terminal fragments that form pores in the plasma membrane and lead to cell lysis [74,89]. This process is accompanied by the release of pro-inflammatory cytokines, including IL-1β and IL-18. In the central nervous system, inflammasomes such as NLRP3 and NLRP1 are involved in pyroptotic signaling. NLRP3 is predominantly expressed in microglia and can be activated by Aβ aggregates, whereas NLRP1 is expressed in neurons and responds to intracellular stress signals [90,91,92]. Activation of these inflammasomes leads to caspase-1 activation and gasdermin-mediated membrane permeabilization.

Inflammasome-driven pyroptosis has been increasingly implicated in AD pathophysiology. Experimental studies have shown that Aβ can activate inflammasome pathways in both neurons and microglia, leading to cytokine release and cell injury [14,73]. Genetic and pharmacological inhibition of inflammasome components, including NLRP3 and caspase-1, has been shown to reduce neuroinflammation and improve cognitive function in AD mouse models [73,93,94]. In human AD brain tissue, increased levels of inflammasome-related markers, such as cleaved caspase-1, ASC complexes, and IL-1β, have been reported [73,95]. In addition, elevated expression of pyroptosis-related proteins, including GSDMD, has been associated with inflammatory responses and disease severity [96,97]. Interactions between pyroptosis and other cell death pathways have also been described. For example, gasdermin E (GSDME) can be cleaved by caspase-3, linking apoptotic signaling to secondary lytic cell death. Experimental studies suggest that Aβ exposure may induce caspase-3/GSDME-dependent pyroptosis-like processes in neuronal models [89]. However, direct evidence of canonical neuronal pyroptosis driven by intracellular tau remains limited. Overall, these findings support a role for pyroptosis in linking neuroinflammation with neuronal injury in AD.

#### 2.2.4. Necroptosis

Necroptosis is a caspase-independent form of programmed cell death characterized by membrane rupture and release of intracellular contents, resulting in a strong inflammatory response [98,99]. It is mediated by a signaling cascade involving receptor-interacting protein kinase 1 (RIPK1), RIPK3, and mixed lineage kinase domain-like protein (MLKL). Under conditions where caspase-8 activity is inhibited, RIPK1 and RIPK3 form a necrosome complex that activates MLKL, leading to membrane disruption [100,101,102]. Emerging evidence suggests that necroptosis is activated in AD and may contribute to neurodegeneration. Increased expression and activation of RIPK1, RIPK3, and MLKL have been observed in postmortem AD brains [15]. These markers have been reported to localize to neurons exhibiting pathological features, including tau-associated structures such as granulovacuolar degeneration bodies, suggesting a link between necroptosis and tau-related pathology [15]. Experimental studies further support a role for necroptosis in AD. Activation of RIPK1-dependent signaling has been associated with neuronal injury and inflammatory responses, while pharmacological inhibition of RIPK1 has been shown to attenuate neuroinflammation and improve functional outcomes in experimental models [103]. In addition, pro-inflammatory cytokines such as TNF-α, which are elevated in AD brains, are known to promote necroptosis under conditions of impaired caspase signaling [98]. These findings suggest that the inflammatory environment in AD may shift cell death pathways toward necroptosis, thereby amplifying neuronal loss.

Although circulating or fluid biomarkers of necroptosis are under investigation, their clinical utility remains uncertain. Moreover, RIPK1 also regulates inflammatory signaling independently of cell death, complicating the interpretation of necroptosis-associated markers [98]. Nevertheless, the convergence of molecular and pathological evidence supports a contributory role of necroptosis in AD pathogenesis. Given its kinase-driven mechanism, necroptosis represents a potential therapeutic target, and inhibitors of RIPK1, RIPK3, and MLKL are being actively explored [103]. Multiple forms of regulated cell death in AD are shaped by converging inflammatory, metabolic, and synaptic disturbances rather than acting as isolated degenerative mechanisms. Chronic neuroinflammation and inflammasome activation may promote neuronal vulnerability, while neuronal injury itself can further amplify inflammatory responses, supporting the existence of bidirectional interactions within the disease network [24,104].

### 2.3. Synaptic Dysfunction

Synaptic dysfunction is a central feature of AD and is closely associated with cognitive decline. Postmortem studies have demonstrated substantial reductions in synapse density in the hippocampus and association cortex, with synaptic loss correlating more strongly with cognitive impairment than amyloid plaque burden or neuronal loss [17,105,106]. Notably, decreased synapse counts are detectable even at early stages, including mild cognitive impairment (MCI), indicating that synaptic pathology precedes widespread neurodegeneration. Consistent with these structural changes, canonical synaptic proteins are reduced in affected brain regions. Synaptophysin, a presynaptic vesicle protein, is decreased in the hippocampus and frontal cortex and serves as a marker of presynaptic terminal loss [107]. PSD-95, a key postsynaptic scaffold protein at excitatory synapses, is also reduced, reflecting dendritic spine degeneration [108]. Lower synaptophysin levels and broader reductions in synaptic protein content have been associated with worse cognitive performance [107,109], while advanced techniques such as array tomography and synaptic proteomics further support widespread downregulation of pre- and postsynaptic proteins [110]. Ultrastructural studies reveal fewer synaptic contacts and reduced synaptic size in AD brains [106]. A major mechanism underlying these changes involves soluble Aβ oligomers, which accumulate at synapses and disrupt receptor signaling and dendritic spine structure [111,112]. Aβ has been shown to interact with synaptic receptors and initiate pathways that promote synapse elimination. In particular, complement proteins such as C1q and C3 localize to synapses early in disease and mediate microglia-dependent synaptic pruning [65]. Genetic or experimental inhibition of complement signaling preserves synapses despite amyloid pathology, supporting a role for complement-mediated elimination in structural synapse loss [65]. Markers of synaptic damage are also detectable in biofluids. Neurogranin, a postsynaptic protein enriched in dendritic spines, is increased in CSF in AD and MCI, consistent with spine degeneration [113]. Similarly, SNAP-25, a presynaptic SNARE protein, is elevated in CSF despite reduced levels in brain tissue [114]. Together, decreased synaptic proteins in brain tissue and increased synaptic markers in CSF provide a coherent signature of synaptic injury. Because these alterations emerge prior to extensive neuronal loss, synaptic biomarkers represent promising tools for disease staging and for monitoring therapeutic interventions aimed at preserving synaptic integrity. Key structural features and associated molecular markers of synaptic dysfunction are summarized in Table 3.

#### Synaptic Plasticity Impairment

Beyond structural synapse loss, AD is characterized by significant impairments in synaptic plasticity, the ability of synapses to strengthen or weaken in response to activity. In hippocampal circuits, soluble Aβ consistently impairs long-term potentiation and, in many experimental systems, facilitates long-term depression [111,119]. These effects are mediated in part by disruption of NMDA- and AMPA-receptor-dependent signaling and downstream pathways that regulate spine function [119]. Brain-derived neurotrophic factor (BDNF) and its receptor TrkB are central regulators of synaptic plasticity. In AD-affected cortex and hippocampus, BDNF levels are reduced, and TrkB signaling is diminished through loss of full-length receptors and increased expression of truncated or cleaved forms. This results in reduced activation of downstream signaling pathways, including PI3K–Akt and ERK/MAPK, which are essential for synaptic maintenance [115,120,121]. Experimental enhancement of TrkB signaling has been shown to restore synaptic plasticity in AD models, supporting BDNF–TrkB signaling as a potential therapeutic target [122].

Activity-dependent signaling pathways are also disrupted. The immediate early gene Arc regulates AMPA receptor trafficking and synaptic remodeling and has been linked to activity-dependent Aβ production [116]. In parallel, CREB signaling is reduced in AD models and the human cortex, contributing to impaired transcription of plasticity-related genes [117,123]. Astrocyte dysfunction may further exacerbate synaptic impairment by reducing glutamate clearance through altered EAAT2/GLT-1 function, thereby promoting excitotoxic stress [118,124]. Markers reflecting these functional abnormalities include reduced phospho-CREB, decreased BDNF and TrkB signaling, and elevated CSF synaptic proteins such as neurogranin, SNAP-25, and synaptotagmin-1 [113,125,126,127]. Because synaptic plasticity deficits precede extensive neuronal loss, preserving synaptic function remains a critical goal for disease-modifying strategies in AD. Synaptic alterations are increasingly recognized as a central interface linking amyloid toxicity, neuroinflammation, and neuronal degeneration in AD. Soluble Aβ species impair synaptic signaling and plasticity, while complement-mediated synaptic pruning driven by activated microglia contributes to progressive synapse loss [65,119]. In addition, synaptic dysfunction may precede overt neurodegeneration, supporting its role as an early contributor to the broader pathological network underlying AD progression [128].

### 2.4. Blood–Brain Barrier Breakdown

AD is associated not only with parenchymal pathology but also with dysfunction of the cerebrovascular system. A key component of this vascular pathology is the BBB, a specialized structure formed by endothelial cells connected by tight junctions and supported by pericytes and astrocytic end-feet [28,129,130]. Accumulating evidence suggests that BBB integrity is compromised in AD, including at early clinical stages [19,28]. Increased BBB permeability has been demonstrated in the hippocampus of individuals with MCI, indicating that vascular dysfunction may occur early in disease progression [19]. Disruption of BBB integrity has multiple consequences. It can impair the delivery of essential nutrients such as glucose, reduce clearance of neurotoxic molecules including Aβ, and allow entry of blood-derived proteins into the brain parenchyma, where they may activate glial cells and contribute to neuronal injury [129,131]. Two major components of BBB dysfunction are particularly relevant in AD: endothelial dysfunction and pericyte degeneration. Cellular components and representative molecular markers associated with BBB disruption are summarized in Table 4.

#### 2.4.1. Endothelial Dysfunction and Tight Junction Loss

In the healthy BBB, endothelial cells are connected by tight junction proteins, including claudin-5, occludin, and zonula occludens-1 (ZO-1), which restrict paracellular permeability and maintain barrier integrity [140,141]. Endothelial cells also express transport systems essential for brain homeostasis. The glucose transporter GLUT1 mediates glucose entry into the brain, while P-glycoprotein (ABCB1) functions as an efflux transporter for substrates including Aβ [134,135]. In AD, structural and functional alterations of endothelial cells have been reported. Postmortem studies have shown reduced and fragmented expression of tight junction proteins such as claudin-5 and occludin in cortical and hippocampal microvessels [142,143]. Ultrastructural analyses further demonstrate widening of inter-endothelial junctions, consistent with impaired barrier integrity [144]. In vivo, dynamic contrast-enhanced MRI studies have revealed increased BBB permeability in patients with MCI, particularly in the hippocampus [19]. Several molecular changes are associated with endothelial dysfunction in AD. Tight junction proteins, including claudin-5, occludin, and ZO-1, are reduced in affected regions [142,143]. Increased expression of adhesion molecules such as VCAM-1 and ICAM-1 has been reported in activated endothelium and is associated with inflammatory signaling pathways relevant to neurovascular dysfunction [132,133]. In addition, reduced GLUT1 expression has been observed in AD brain tissue and is associated with impaired cerebral glucose transport [136]. Decreased activity of P-glycoprotein has also been reported and may contribute to reduced clearance of Aβ from the brain [145].

Direct evidence of BBB leakage has been observed in AD brain tissue, where blood-derived proteins such as fibrinogen accumulate in perivascular regions [28,137,146]. Consistently, an increased CSF to serum albumin ratio has been reported in AD patients, reflecting impaired BBB integrity [138]. Together, these findings indicate that BBB dysfunction in AD is associated with increased permeability and impaired clearance capacity.

#### 2.4.2. Pericyte Degeneration and Vascular Support Failure

Pericytes surround brain capillaries and play essential roles in BBB stability, regulation of cerebral blood flow, and clearance of solutes along perivascular pathways. In AD, pericyte injury and loss have been identified as early vascular abnormalities, with BBB dysfunction particularly evident in APOE4 carriers [18,139,147,148]. Loss of pericyte support compromises vascular integrity, impairs cerebral perfusion, and may reduce Aβ clearance [18,147]. Markers of pericyte injury include reduced coverage of microvessels by PDGFRβ-positive cells and increased levels of soluble PDGFRβ in CSF, which reflects pericyte damage [139,148]. Elevated CSF sPDGFRβ has been associated with increased BBB permeability measured by imaging and with cognitive decline in longitudinal studies [18,148]. Functionally, pericyte loss contributes to reduced capillary perfusion and impaired neurovascular responses, leading to chronic hypoperfusion and metabolic stress in neural tissue [147]. Pericyte dysfunction has also been linked to alterations in basement membrane composition and impaired perivascular clearance pathways [131,149]. At the level of the neurovascular unit, disrupted signaling among neurons, astrocytes, endothelial cells, and pericytes contributes to neurovascular uncoupling [150,151]. Vascular risk factors such as hypertension and diabetes may exacerbate these abnormalities. These observations are consistent with the two-hit vascular hypothesis, in which vascular dysfunction promotes amyloid-related and downstream neurodegenerative changes [131]. Beyond vascular injury itself, BBB disruption may influence multiple downstream mechanisms involved in AD progression. Impaired barrier integrity can reduce the clearance of Aβ and other metabolites while facilitating the entry of blood-derived factors that promote neuroinflammatory responses and neuronal stress [18,152]. These vascular alterations may further interact with glial activation and synaptic dysfunction, reinforcing the interconnected nature of AD pathology [153].

### 2.5. Myelin Damage and White Matter Disruption

Although AD has traditionally been considered a gray matter disorder, increasing evidence indicates that white matter is also affected [154]. Myelin damage and axonal degeneration have been reported in AD and may occur early in the disease process, in some cases preceding overt gray matter atrophy [155,156,157]. These alterations disrupt structural connectivity and are associated with cognitive impairment, as intact myelin is essential for efficient signal conduction [156,158]. White matter pathology in AD involves both oligodendrocyte dysfunction with demyelination and axonal degeneration, which are closely interconnected processes [157,159]. Major pathological features and associated molecular markers of myelin and axonal damage are summarized in Table 5.

#### 2.5.1. Oligodendrocyte Injury and Demyelination

Oligodendrocytes are responsible for the production and maintenance of myelin [162]. In AD, both neuroimaging and neuropathological studies support the presence of oligodendrocyte dysfunction and myelin injury [155,163]. MRI studies frequently reveal white matter hyperintensities and diffusion abnormalities, consistent with reduced white matter integrity [164]. Postmortem analyses have demonstrated myelin sheath disruption, altered expression of myelin-associated proteins, and oligodendrocyte alterations in affected regions [163]. Myelin-related proteins such as MBP, proteolipid protein 1 (PLP1), and MAG are commonly used to assess myelin pathology. The MAG:PLP1 ratio has been proposed as an indicator of hypoperfusion-related myelin injury in AD [165]. Experimental studies further suggest that MBP can interact with Aβ and influence its aggregation or degradation [166]. White matter abnormalities are also observed in presymptomatic individuals carrying familial AD mutations, indicating that myelin-related pathology may arise early in disease progression. In these populations, white matter changes have been associated with CSF Aβ and tau measures and with subsequent clinical decline [167].

#### 2.5.2. Axonal Damage and Cytoskeletal Pathology

In addition to myelin pathology, axonal injury is an important component of AD. Tau hyperphosphorylation destabilizes microtubules and disrupts axonal transport, leading to axonal swellings with accumulation of transported cargo, including amyloid precursor protein (APP) and organelles [160,168]. APP-positive axonal spheroids, particularly in proximity to amyloid plaques, are commonly observed and are indicative of axonal injury [160]. Alterations in neurofilament proteins have been reported in damaged neurons and dystrophic neurites in AD, as demonstrated in neuropathological studies [169,170]. In addition, NfL in CSF and plasma has emerged as a sensitive biomarker of ongoing axonal degeneration and disease progression [161]. Disruption of axonal transport contributes to the accumulation of cytoskeletal proteins, including tau and neurofilaments, within damaged axons [168]. Secondary axonal degeneration, including Wallerian-like processes, may occur in association with neuronal loss and demyelination, contributing to white matter tract degeneration and cognitive impairment [158,171].

Oligodendrocytes provide metabolic support to axons, and myelin damage may increase axonal vulnerability. Conversely, axonal injury can impair oligodendrocyte function, contributing to a cycle of degeneration [172]. In addition, neuroinflammatory processes may further exacerbate white matter injury, as microglial activation has been associated with impaired remyelination and oligodendrocyte precursor cell differentiation [173]. Clinically, white matter and axonal injury have been associated with slowed processing speed and executive dysfunction in AD [158,174]. Increasing evidence suggests that white matter pathology not only reflects downstream neurodegeneration but may also actively contribute to network dysfunction and cognitive decline in AD [21]. Myelin damage and axonal injury may arise from chronic neuroinflammation, vascular dysfunction, and metabolic stress, while disrupted structural connectivity may further exacerbate neuronal vulnerability and disease progression [21,153].

## 3. Integrated Temporal and Systems-Level Framework of AD Pathology

**Figure 2:**
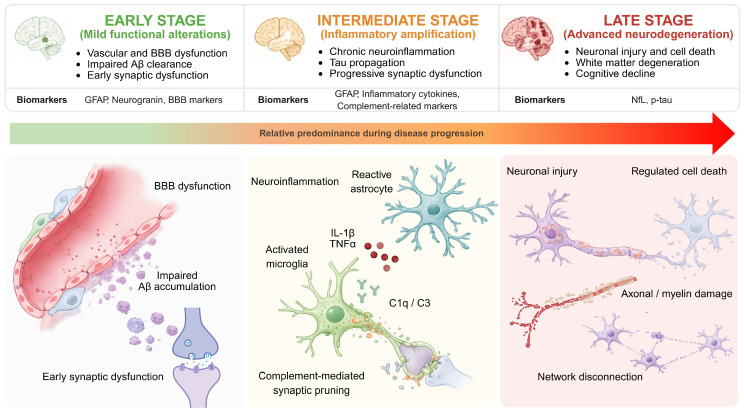
Integrated temporal and systems-level framework of AD pathology. Conceptual illustration of the interacting pathological processes during AD progression. Early-stage functional alterations include BBB dysfunction, impaired Aβ clearance, and synaptic dysfunction, which may contribute to glial activation and neuroinflammatory amplification. Sustained neuroinflammation and complement-mediated synaptic pruning may further promote neuronal stress and progressive synaptic dysfunction. In later stages, neuronal injury, regulated cell death, axonal/myelin damage, and network disconnection become more prominent and contribute to cognitive decline. Representative stage-associated biomarkers related to functional alterations, neuroinflammation, and neurodegeneration are also summarized.

AD progression is increasingly recognized as a consequence of interacting disease mechanisms involving vascular dysfunction, impaired protein clearance, glial activation, synaptic dysfunction, neuronal injury, and white matter degeneration [175,176]. Although the temporal hierarchy of these alterations remains incompletely understood, accumulating evidence suggests that specific mechanisms may become relatively more prominent during different phases of disease progression rather than following a strictly linear sequence [39,176]. Therefore, the stage-related descriptions presented here should be interpreted as a conceptual framework for understanding the interacting and overlapping mechanisms underlying AD progression (Figure 2).

From a temporal perspective, early-stage AD is increasingly associated with functional alterations rather than overt neurodegeneration [39,176]. In particular, vascular dysfunction and BBB disruption may impair the clearance of Aβ and other metabolites, thereby facilitating their accumulation within the brain [18,152]. In parallel, synaptic dysfunction, including impaired synaptic plasticity and early synapse loss, is increasingly recognized as an early feature of AD that may precede substantial neuronal loss [119]. These early functional disturbances may increase vulnerability to subsequent neurodegenerative amplification [177]. These early alterations are closely linked to glial activation and neuroinflammation. Microglia and astrocytes respond to Aβ accumulation, vascular dysfunction, and synaptic stress by adopting activated phenotypes and releasing cytokines, chemokines, and complement components [9,24]. Although these responses may initially support protective clearance mechanisms, sustained glial activation can become maladaptive, promoting complement-mediated synaptic pruning, chronic inflammation, and progressive neuronal stress [65]. Accordingly, neuroinflammation may function as a central mediator linking early functional disturbances to progressive neurodegeneration [24].

During intermediate phases of disease progression, pathological tau accumulation and propagation may further contribute to synaptic dysfunction, neuronal vulnerability, and large-scale network disruption, thereby promoting progressive neurodegeneration [178,179]. In addition, tau pathology may interact with inflammatory and vascular alterations, further amplifying neuronal dysfunction and pathogenic signaling cascades [8,180]. As the disease progresses, the cumulative effects of chronic inflammation, synaptic dysfunction, and metabolic stress may contribute to neuronal injury and regulated cell death [25]. In parallel, axonal injury and white matter degeneration, including myelin disruption and impaired axonal transport, may further exacerbate network disconnection and cognitive decline [21]. These later-stage alterations are often associated with more pronounced neurodegeneration and clinical deterioration, although the extent and timing of these processes may vary substantially across individuals.

Importantly, the relative contributions and temporal relationships among Aβ pathology, neuroinflammation, vascular dysfunction, and tau-associated neurodegeneration remain incompletely resolved and may differ across patient populations [8]. While some models propose that Aβ accumulation represents an early initiating event, others emphasize vascular dysfunction or neuroinflammatory processes as key drivers or amplifiers of disease progression [152]. Therefore, AD is more appropriately conceptualized as a dynamic and heterogeneous network of interacting mechanisms rather than a strictly linear pathological cascade [176]. This perspective highlights the importance of integrative approaches for understanding disease progression and identifying stage-specific therapeutic targets.

## 4. Clinical Implications and Biomarker Integration in AD

The integration of functional pathological processes with molecular biomarkers has important implications for clinical diagnosis, prognosis, and disease monitoring in AD. In recent years, fluid biomarkers derived from CSF and plasma have emerged as key tools for assessing disease-related changes in vivo [39]. Core biomarkers reflecting Aβ and tau pathology, including the Aβ42/40 ratio and phosphorylated tau species (p-tau181 and p-tau217), are widely used for diagnostic classification and disease staging [39]. In particular, plasma p-tau217 has demonstrated high diagnostic accuracy for distinguishing AD from other neurodegenerative disorders and shows strong concordance with amyloid and tau PET imaging [181]. In addition to these core biomarkers, markers reflecting functional pathological processes provide complementary information about disease activity. NfL, measured in CSF or plasma, is a well-established marker of axonal injury and neurodegeneration and is associated with disease progression and clinical severity [182]. GFAP, an astrocytic marker, reflects astrocyte activation and increases early in the disease course, including during preclinical stages [183]. Markers of synaptic dysfunction, such as neurogranin in CSF, are associated with synaptic loss and cognitive decline [184]. Similarly, biomarkers of vascular dysfunction, including the CSF-to-serum albumin ratio and measures of BBB permeability, provide insight into neurovascular alterations in AD [18].

Importantly, the relative informativeness of these biomarkers depends on the clinical context and disease stage. Amyloid and tau biomarkers are particularly informative for diagnosis and early disease detection, whereas markers such as NfL and synaptic proteins are more closely associated with neurodegeneration and clinical progression [39]. Glial and vascular biomarkers may capture early functional alterations and ongoing pathological activity, thereby providing additional value for disease monitoring and therapeutic evaluation [18,183]. Moreover, considerable heterogeneity exists among patients with AD, both in terms of pathological features and biomarker profiles. Differences in genetic background, vascular risk factors, and comorbid conditions may influence the relative contributions of amyloid pathology, neuroinflammation, and vascular dysfunction [176]. As a result, individual patients may exhibit distinct biomarker patterns, highlighting the need for multimodal and integrative approaches to disease characterization.

Taken together, the combined assessment of core proteinopathy markers and functional pathology-based biomarkers provides a more comprehensive framework for understanding disease progression. Such an approach may improve diagnostic accuracy, enable more precise disease staging, support personalized therapeutic strategies targeting specific pathological processes, and facilitate patient stratification and treatment monitoring in emerging disease-modifying clinical trials [185].

## 5. Conclusions

AD is increasingly recognized as a multifactorial and heterogeneous neurodegenerative disorder in which classical proteinopathies involving Aβ and tau interact with a range of functional pathological processes. This review highlights that neuroinflammation, regulated neuronal cell death, synaptic dysfunction, BBB disruption, and white matter injury are not isolated events but rather interconnected mechanisms that collectively shape disease progression. At the cellular level, dynamic interactions among microglia, astrocytes, neurons, endothelial cells, and oligodendrocytes contribute to a complex pathological landscape that evolves throughout the course of AD. These interactions are reflected in distinct molecular and fluid biomarkers that provide insight into disease mechanisms and offer opportunities for biomarker development. Importantly, many of these functional alterations emerge during early stages of disease progression, often before substantial neuronal loss becomes apparent, underscoring their relevance for early diagnosis and intervention. The integration of these functional pathological features provides a more comprehensive framework for understanding AD beyond the traditional amyloid- and tau-centered perspective. In particular, the identification of cell-type-specific markers and pathway-associated biomarkers may improve disease characterization, support more precise patient stratification and facilitate the development of targeted therapeutic strategies.

Future research should focus on further elucidating the temporal and mechanistic relationships among vascular dysfunction, neuroinflammation, synaptic injury, and neurodegeneration, while also addressing the substantial heterogeneity observed across patient populations. Advances in multi-omics approaches, neuroimaging, and fluid biomarker analysis will be critical for capturing the dynamic complexity of AD pathophysiology in vivo. Ultimately, a systems-level understanding integrating molecular, cellular, vascular, and network-level alterations will be essential for developing effective disease-modifying therapies. In conclusion, AD should be viewed as a disorder of interacting pathological mechanisms in which inflammation, neurodegeneration, vascular dysfunction, and connectivity loss converge. Targeting these interconnected processes represents a promising direction for improving diagnosis, monitoring disease progression, and developing more effective therapeutic interventions.

## Figures and Tables

**Table 1:** Cell-type-specific states and representative markers of neuroinflammation in AD.

Cell Type	State/Subtype	Cellular Markers	Pathway Markers	Reference
Microglia	Homeostatic	P2RY12, TMEM119, Iba1, CX3CR1	-	[22,49]
	Activated	CD68, HLA-DR	IL-1β, TNF-α	[22,41,50]
	Disease-associated microglia (DAM)	TREM2, APOE, ITGAX	Phagocytosis- related pathways	[44,45]
Astrocytes	Reactive	GFAP, vimentin	-	[46]
	A1-like	GFAP, C3	IL-1α, TNF-α	[10,41,51]
	Disease-associated astrocyte (DAA)	GFAP, C3, Serpina3n	Complement- related pathways	[52]

**Table 2:** Major regulated cell death pathways and representative markers in AD.

Cell Death Type	Key Markers	Reference
Apoptosis	Cleaved caspase-3, BAX, BAD	[16,70]
Ferroptosis	GPX4, SLC7A11, Ferritin	[71,72]
Pyroptosis	Caspase-1, GSDMD, NLRP3	[73,74]
Necroptosis	RIPK1, RIPK3, MLKL	[15]

**Table 3:** Key structural features and associated markers of synaptic dysfunction in AD.

Synaptic Feature	Key Markers	Reference
Synapse loss	Synaptophysin, SNAP-25, PSD-95, Neurogranin	[26,107]
Synaptic pruning	C1q, C3	[65]
Plasticity impairment	BDNF, TrkB, Arc, CREB	[115,116,117]
Excitotoxic stress	EAAT2 (GLT-1)	[118]

**Table 4:** Cellular components and molecular markers of blood-brain barrier (BBB) breakdown in AD.

BBB Component/Feature	Key Markers	Reference
Tight junction disruption	Claudin-5, Occludin, ZO-1	[130]
Endothelial activation	VCAM-1, ICAM-1	[132,133]
Transport dysfunction	GLUT1, ABCB1 (P-gp)	[134,135,136]
Barrier leakage	Fibrinogen, albumin	[137,138]
Pericyte degeneration	PDGFRβ	[139]

**Table 5:** Pathological features and molecular markers of myelin and axonal damage in AD.

Pathologic Feature	Key Markers	Reference
Demyelination	MBP, PLP1, MAG	[21,154]
Oligodendrocyte dysfunction	OLIG2, MOG	[21,30]
Axonal injury	APP	[160]
Cytoskeletal damage	NfL	[161]

## Data Availability

Not applicable.
